# Immobilization of Cross-Linked Phenylalanine Ammonia Lyase Aggregates in Microporous Silica Gel

**DOI:** 10.1371/journal.pone.0080581

**Published:** 2013-11-15

**Authors:** Jian Dong Cui, Lian Lian Li, Hong Jie Bian

**Affiliations:** 1 Research Center for Fermentation Engineering of Hebei, College of Bioscience and Bioengineering, Hebei University of Science and Technology, Shijiazhang, P R China; 2 National Key Laboratory of Biochemical Engineering, Institute of Process Engineering, Chinese Academy of Sciences, HaiDian District, Beijing, P R China; 3 Key Laboratory of Industry Microbiology, Ministry of Education, Tianjin University of Science and Technology, Tai Da Development Area, Tianjin, P R China; Queen's University Belfast, United Kingdom

## Abstract

A separable and highly-stable enzyme system was developed by adsorption of phenylalanine ammonia lyase (PAL) from *Rhodotorula glutinis* in amino-functionalized macroporous silica gel and subsequent enzyme crosslinking. This resulted in the formation of cross-linked enzyme aggregates (PAL-CLEAs) into macroporous silica gel (MSG-CLEAs). The effect of adsorptive conditions, type of aggregating agent, its concentration as well as that of cross-linking agent was studied. MSG-CLEAs production was most effective using ammonium sulfate (40%-saturation), followed by cross-linking for 1 h with 1.5% (*v/v*) glutaraldehyde. The resulting MSG-CLEAs extended the optimal temperature and pH range compared to free PAL and PAL-CLEAs. Moreover, MSG-CLEAs exhibited the excellent stability of the enzyme against various deactivating conditions such as temperature and denaturants, and showed higher storage stability compared to the free PAL and the conventional PAL-CLEAs. Such as, after 6 h incubation at 60°C, the MSG-CLEAs still retained more than 47% of the initial activity whereas PAL-CLEAs only retained 7% of the initial activity. Especially, the MSG-CLEAs exhibited good reusability due to its suitable size and active properties. These results indicated that PAL-CLEAs on MSG might be used as a feasible and efficient solution for improving properties of immobilized enzyme in industrial application.

## Introduction

Biocatalytic processes are more environmentally friendly, more cost-effective and more sustainable. Consequently, in the last two decades, the biocatalysis has emerged as an important technology for meeting the growing demand for green and sustainable chemicals manufacture [1], [2]. However, only in very few cases will a biocatalytic transformation on a technical scale work perfectly at the first attempt because of limitations related to their poor reusability and low operational stability. To overcome these limitations, many carrier immobilized enzyme strategies are attempted to improve a biocatalytic process for optimal product yields, such as physical adsorption, multipoint covalent attachment and encapsulation with materials [3]–[5].

Recently, a new carrier-free immobilized enzyme strategy, cross-linked enzyme aggregates (CLEAs), was first demonstrated in the 2000s [6], and has attracted increasing attention. By cross-linking the physical enzyme aggregates which are of supramolecular structures, enzyme can be simply immobilized with high stability and high volume activity. However, CLEAs has also some undesirable properties, such as, CLEAs are fragile for many industrial applications in almost any kind of reactor configuration (basket reactors may be an exception) and it is difficult to handle and fully recover the CLEA particle [7]–[9]. Moreover, the small particle of CLEAs (below 10 µm) might form increased clumps by centrifugation and filtration treatments, which would hamper CLEAs to disperse again in solution and thereby result in low catalytic efficiency [10]. In order to improve these drawbacks, in recent years, some researchers have developed supported CLEAs strategies as alternative solutions to get a biocatalyst with good mechanical properties [11]–[13]. The macroporous silica gel is usually used as the catalyst carrier because of their controlled porosity and high surface area. Kim et al. reported the immobilization of α-chymotrypsin through cross-linking into hierarchically ordered mesoporous silica (HMMS). CLEA of a-chymotrypsin (CLEA-CT) in HMMS showed a high enzyme loading capacity and significantly increased enzyme stability [14]. β-glucosidase was immobilized onto mesocellular silica foams (MCF) by using enzyme adsorption followed by glutaraldehyde (GA) crosslinking. The resulting crosslinked enzyme aggregates (CLEAs) showed an impressive stability with high enzyme loadings [15]. However, the mesocellular pore size was very small (about 30 nm) which limited the maximum loading of CLEAs to a certain degree. Consequently, it is beneficial to immobilize CLEAs in microporous silica gel.

Phenylalanine ammonia-lyase (E.C.4.3.1.5-PAL) belongs to the family of lyases [16], [17]. It has been used chiefly in the manufacture of L-phenylalanine by reversing the enzyme reaction with high concentration of trans-cinnamic acids and ammonia at an elevated pH. However, the production of L-phenylalanine from trans-cinnamic acids was of limited success, partly because of the relatively low specific activity and instability of PAL during the bioconversion [18], [19]. In order to overcome these problems, recently, the preparation of cross-linked enzyme aggregates of crude PAL from *Rhodotorula glutinis* (*R. glutinis*) (PAL-CLEAs) was first introduced in our group. Compared to the free enzyme, the PAL-CLEAs exhibited the increased stability of the enzyme against various deactivating conditions such as pH, temperature, denaturants, and organic solvents [20]. However, one of the undesirable properties of PAL-CLEAs is that their particle size is small. As a result, it is difficult to isolate and recover CLEAs from the reaction system only by centrifugation or filtration. Consequently, the present work explores the feasibility of developing a facile method for preparing a new kind of PAL-CLEAs through cross-linking PAL into the pores of the amino modified macroporous silica gel (MSG-CLEAs). The effects of adsorption, precipitation and cross-linking on the enzyme activity were investigated. The optimal catalytic temperature and pH as well as the stability of MSG-CLEAs were measured. Lastly, the reusability of MSG-CLEAs was also measured.

## Materials and Methods

### Chemicals

Glutaraldehyde was obtained from Sigma-Aldrich Inc. (St. Louis, MO, U.S.A.). Macroporous silica gel (MSG, with diameter of 1 mm) was purchased from MaKall group co., Itd (QingDao, China). All other reagents were of analytical grade.

### Production of PAL in *R. glutinis*

The strain of *R. glutinis* (CICC 32917) used throughout this study was obtained from China Center of Industrial Culture Collection (CICC, Beijing, China). The strain was maintained on potato-glucose agar slant at 4°C. The medium for seed culture consisted of (g/L) glucose 5, peptone 10, yeast extract 10, NaCl 5 and KH_2_PO_4_ 1, pH 5.5. The PAL production medium (g/L) included glucose 1, peptone 35, NaCl 5, KH_2_PO_4_ 0.25 and (NH_4_)_2_HPO_3_ 1.5, pH 5. For seed preparation, the cells on the slant stock medium were cultivated for 3 days at 30°C, and were inoculated into 50 ml seed medium in a 250 Erlenmeyer flask and cultivated on a 150 rpm reciprocal shaker for 20 h at 30°C. Then the 10% (v/v) seed culture was transferred to 50 mL of production medium in a 250 ml flask, and the flask was incubated at 30°C with shaking at 150 rpm for 72 h. The culture broth was centrifuged at 6000 ×g for 10 min at 4°C. The supernatant was removed. After washing with 0.9% sterilized saline and water, the cells were resuspended in a potassium phosphate buffer (10 mM, pH 7.5). Then, the cells were disrupted by intermittent ultrasonication, the cell extracts were centrifuged at 10,000×g for 15 min. Solid (NH_4_)_2_SO_4_ was added to bring the supernatant to 55% saturation and centrifuged (10,000×g) after 30 min. The precipitate was dissolved in 25 mM-Tris-HCI buffer, pH8.8, and centrifuged. The clear supernatant (10 ml) was loaded on a column (15 cm ×1 cm) of ion exchange chromatography in DEAE-Sephacel pre-equilibrated with the same buffer. The enzyme was eluted with a linear gradient of 75 ml of 25 mM-Tris-HCI buffer, pH8.8, and 75 ml of the same buffer containing 300 mM-NaCl. The active fractions from the DEAE-Sephacel column (Beijing DingGuo ChangSheng Biotechnology Co., Ltd, China) were pooled, and the partially purified PAL was obtained and utilized for the preparation of CLEAs.

### Surface modification

The surface of the MSG was coated with 3-aminopropyl triethoxysilane (APTES) by a silanization reaction in order to obtain amino modified MSG. The procedure was carried out according to previous report [7]. MSG was pre-purified with hydrochloric acid (20%, *v/v*) for 8 h, washed with distilled water, and dried at 120°C for 12 h. These dried MSG (4 g) were added to the modifying regent (APTES/toluene, 1/15, *w/v*). The mixture was refluxed for 7 h, and the toluene was evaporated under vacuum. The amino modified MSG was washed with distilled water and dried at 100°C. The content of amino-group was determined by salicylaldehyde [21]. FTIR spectra for the modified MSG were obtained with a NEXUS870 infrared spectrometer (Thermo Nicolet Corporation, USA) using the standard KBr disk method.

### Preparation of CLEAs and MSG-CLEAs of PAL

The CLEAs of PAL were prepared by conventional method. PAL was dissolved in 25 mM Tris-HCl buffer (pH 8.8). Different concentration of ammonium sulphate or PEG400 were added dropwise to the PAL solution under gentle stirring at 25°C for 1 h. Glutaraldehyde (GA) was added and stirred for 1 h at 25°C., and then centrifuged at 10000×g for 10 min. Afterwards, the pellet was washed for 3 times with 25 mM Tris-HCl buffer (pH 8.8) and finally stored in the same buffer at 4°C.

For MSG-CLEAs, the different amount of the amino modified MSG (g) was mixed with free PAL (mL) in 25 mM Tris-HCl buffer solution (pH 8.8) and shaken for 2.5 h at 25°C. Then the samples were washed by 0.2 M phosphate buffer (pH 7.0) and added intodifferent concentration of ammonium sulphate or PEG400 with shaking to precipitate the free PAL for 1 h. Afterwards, the different concentrations of GA (0.1%, 0.5%, 1%, 1.5%, 2%, 2.5% and 3%) were added in the mixture and shaken for 1 h, and then centrifuged at 10000×g for 10 min. MSG-CLEAs were washed for 3 times with 0.2 M phosphate buffer (pH 7.0) and finally stored in the same buffer at 4°C.. Herein, the preparing conditions of MSG-CLEAs have been optimized through single factor experiment.

### Enzyme activity assay

The activity of free PAL, PAL-CLEAs and MSG-CLEAs were determined by the modification of the procedure [19], [20]. The reaction mixture (5 mL) containing 25 mM Tris–HCl buffer (pH 8.8), 25 mM L-Phenylalanine and a amount of enzyme samples were incubated at 30°C for 20 min. The reaction was terminated by addition of 1 M HCl. After centrifugation, the absorbance of the clear supernatant was measured at 280 nm with a 752 spectrophotometer (Shanghai Precision and Scientific Instrument Co., China). One unit of PAL activity was defined as the amount of enzyme required to convert one μmole of L-phenylalanine to trans-cinnamic acids per min under above conditions. The activity recovery in CLEAs was calculated as given in Eq. (1):


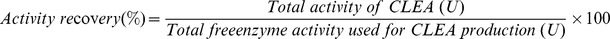
 The relative activity (%) was calculated by the ratio of the residual activity to the initial activity. PAL activity (U/g) is expressed as units of enzyme per gram of MSG.

### Structural characterization by scanning electron microscopy

Scanning electron micrographs (SEMs) of MSG-CLEAs were recorded on Hitachi S-4800 FESEM (Hitachi Co. Ltd, Japan) electron microscopes. The samples were dried at ambient temperature and then coated with platinum using an Emitech K550 (Ashford, UK) sputter coater.

### Optimal conditions for enzyme activity

The optimal temperature of the free PAL, PAL-CLEAs and MSG-CLEAs were determined by adding the enzyme samples into the substrate solution (pH 8.8) at different temperatures (30–60°C) for 30 min, and the optimal pH was determined by adding the enzyme samples into the substrate solution of different pH 6, 7, 8, 8.8, 9.5 and 10.5 at 30°C for 30 min.

### The stability of free enzyme, PAL-CLEAs and MSG-CLEAs

The thermal stability of free-PAL, PAL-CLEAs and MSG-CLEAs was evaluated by incubating enzyme samples in 0.2 M phosphate buffer (pH 7.0) without substrate at 60°C for 1-6 h. At different incubation times, PAL activity was determined by the same procedure as described above. The stability against different chemical denaturants was tested. The denaturing solutions consisted of urea (6 M), sodium dodecyl sulfate (SDS, 2%, w/v) or ethanol (40%, v/v) in 0.2 M phosphate buffer (pH 7.0). The tests lasted 30 min at 30°C. Storage stabilities of the free PAL, PAL-CLEAs and MSG-CLEAs were determined by incubating enzyme samples in 0.1 M phosphate buffer (pH 7.0) without substrate at 25°C. At different storage times, free PAL, PAL-CLEAs and MSG-CLEAs were separated and washed by distilled water. Then the PAL activity in these immobilized enzyme and free enzyme samples was determined.

### Determination of kinetic parameters

The kinetic constants (*K*_m_ and *V*_max_ values) for the free PAL, PAL-CLEAs and MSG-PAL PAL were determined by measuring the enzymatic reaction rates (using the same amount of enzyme) at different substrate concentrations (0.5–2.5 mM). *K*_m_ and *V*_max_ were calculated from the Lineweaver-Burk equation using computed linear regression calculations.

### Reusability of PAL-CLEAs and MSG-CLEAs

Reusability of PAL-CLEAs and MSG-CLEAs was evaluated in batch operation mode in the biotransformation of L-phenylalanine reaction. The reaction using 50 mg CLEAs and 10 g/L trans-cinnamic acid in 28% ammonium hydroxide (pH 10.0) was carried out at 30°C for 2 h. After each cycle, PAL-CLEAs and MSG-CLEAs were separated using centrifugation separation, respectively, washed with buffer and then suspended again in a fresh reaction mixture to measure enzyme activity. The PAL activity of each cycle was determined in terms of residual activity by taking the enzyme activity of the first cycle as 100%.

## Results and Discussion

### Surface modification of MSG for CLEAs-MSG preparation

**Figure 1:**
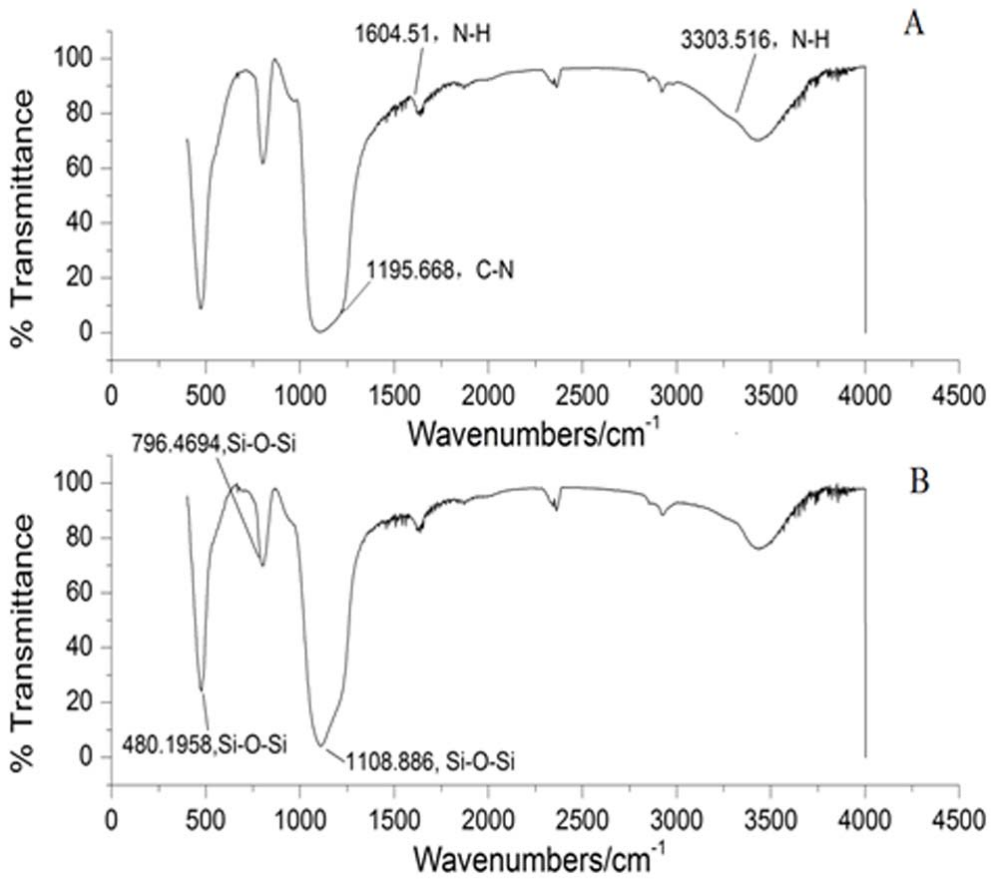
FTIR spectra of MSG, (A) MSG with surface modification; (B) MSG without surface modification.

Fig. 1 presented the FTIR spectra of MSG and modified MSG samples. The FTIR transmittance spectra were recorded in the region of 400–4000 cm^−1^. The typical Si-O-Si bands around 1108, 796 and 480 cm^−1^ were observed on all samples [22], and the band assigned to C-N was observed at 1195 cm^−1^. The band assigned to N-H was observed at 1604 cm^−1^ and 3303 cm^−1^, respectively. The content of amino group was 480 µmol/g, while it could not be determined before modification. These results suggested that the amino-modification in the MSG was occurred.

### Enzyme adsorption

**Figure 2:**
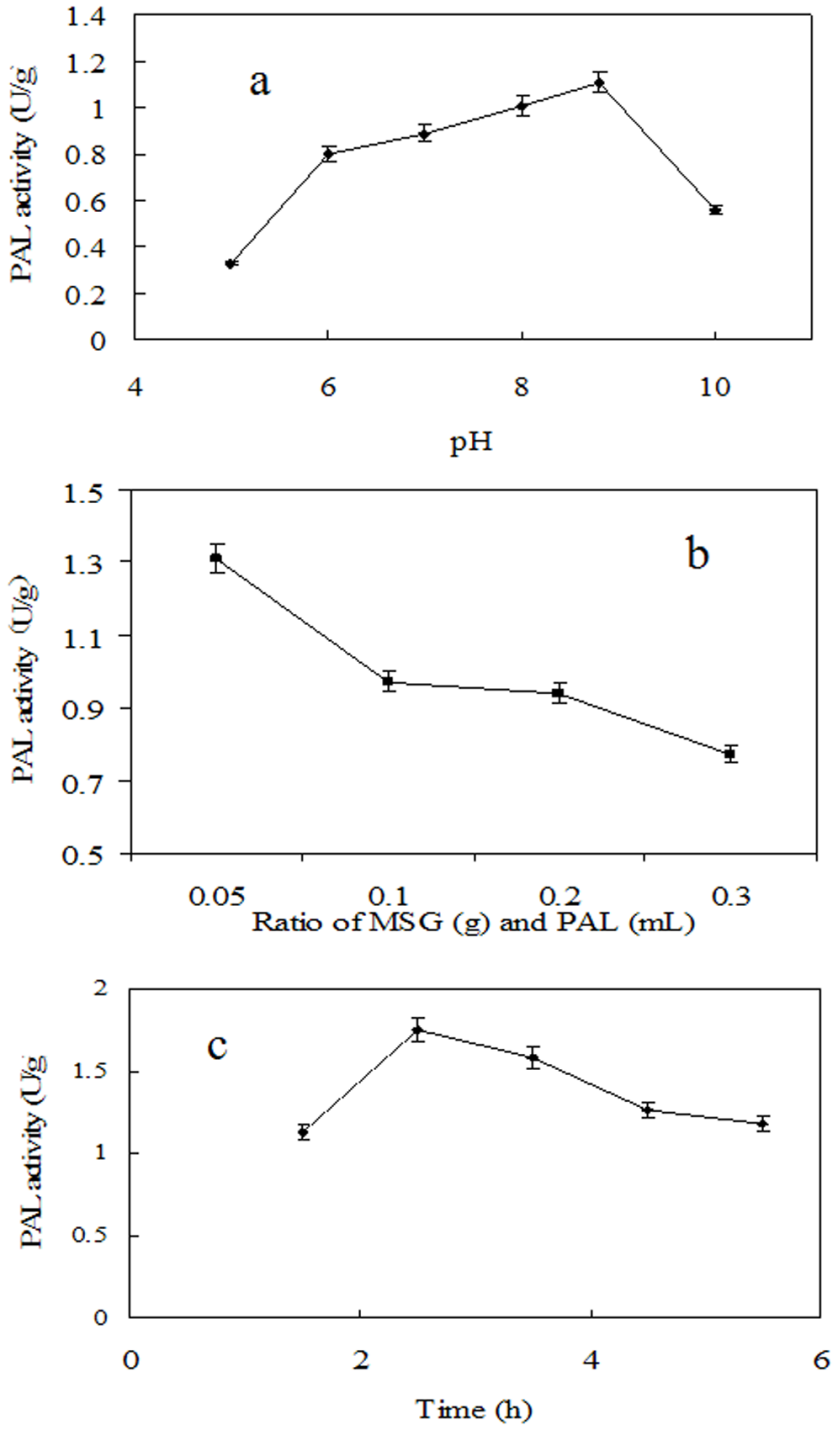
The effects of (a) enzyme solution pH; (b) the ratio of enzyme and silica gel; (c) adsorption time on the enzyme activity.

**Figure 3:**
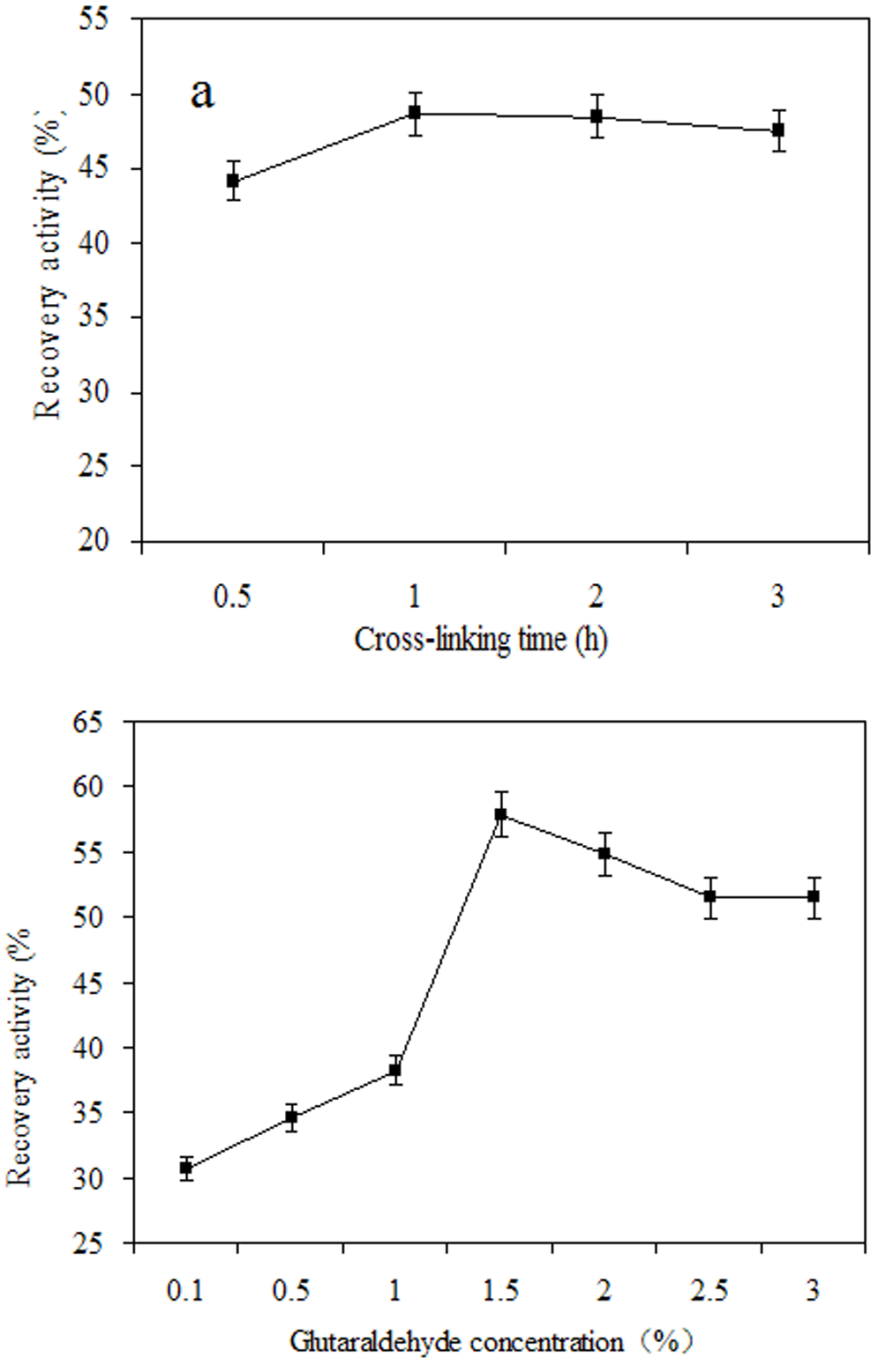
The effects of cross-linking time (a) and GA concentration (b) on the activity of MSG-CLEAs.

Generally, Physical adsorption is one of the simple and straightforward routes for enzyme immobilization, in which the enzyme is bound to a support by hydrophobic, van der Waals or ionic interactions. It is often used because of the ease and low cost of the procedure [25]. These interactions can be influenced by the pH of the solution, the ratio of enzyme and carriers, and the adsorption time. Consequently, in this study, the effect of the pH of the solution, the ratio of enzyme and carriers, and the adsorption time on the activity in adsorption step was first studied. As shown in Fig. 2a, PAL activity was affected significantly by the pH of the solution, the activity enhanced with increasing pH, the highest activity was found to be at pH 8.8. Furthermore, PAL activity also depended on the ratio of enzyme and carriers. Fig. 2b indicated that activity rose with the decrease of the ratio of enzyme and carriers. The highest activity was obtained when the ratio of enzyme and carriers was 0.05 g/mL. which indicated that the enzymes embedded in the MSG were saturated at this concentration. In addition, adsorption time also exhibited remarkable effects on activity, as showed in Fig. 3c, The highest activity was observed at 2.5 h, however, after 2.5 h, the activity decreased with increasing adsorption time. Therefore, the adsorption time was controlled at about 2.5 h to obtain the highest.

### Preparation of MSG-CLEAs

The precipitants were added after free PAL had been adsorbed on MSG. Generally, the precipitant has an important effect on the activity recovery in CLEAs as it causes physical aggregation of enzyme molecules into supramolecular structures [23], [24]. Consequently, it is necessary to screen a number of precipitants for CLEAs preparation. Table 1 shows activity recovery after 1 h of precipitation using different concentration of ammonium sulfate or PEG400. The results showed that the activity recoveries were relatively high when ammonium sulfate was used as precipitants. Moreover, the best results (recovery of about 95% of the initial PAL activity) were obtained using 40% (w/v) of ammonium sulfate as precipitant. So the cheap ammonium sulfate was selected as the optimized precipitating solvent for further investigation. In addition, different concentrations of glutaraldehyde (GA) were tried for the cross-linking of the aggregates to achieve the maximum activity recovery in MSG-CLEAs. Maximum activity recovery (60%) was observed with 1.5% (*v/v*) (Fig. 3a). Further increase in glutaraldehyde concentration above 1.5% resulted in small decrease in the activity recovery. The small decrease in the activity recovery could possibly be due to an excessive cross-linking of enzyme molecules making them catalytically inactive. Moreover, effect of cross-linking time on the activity recovery of MSG-CLEAs was also studied. The results showed that the increase in cross-linking time resulted in slight increase in the activity recovery. The cross-linking reached to optimum within 1 h where maximum activity recovery was obtained (Fig 3b). Beyond 1 h, small decrease in the activity recovery was observed. The previous reports showed that the activity recovery and the aggregation yield increase with extent of cross-linking. However, additional cross-linking leads to decrease in the activity recovery [7], [20]. Too much cross-linker can result in a loss of the minimum flexibility needed for the activity of enzyme while too little cross-linker is used the enzyme molecule may still be too flexible.

**Table 1:** The effect of precipitants on PAL activity.

precipitants	Specific PAL activity (U/g)	Recovery activity (%)
No precipitants	0.1473	100
20% (NH_4_)_2_SO_4_	0.1387	94
40% (NH_4_)_2_SO_4_	0.1397	95
60% (NH_4_)_2_SO_4_	0.1296	88
80% (NH_4_)_2_SO_4_	0.0997	68
100% (NH_4_)_2_SO_4_	0.1113	76
40%PEG400	0.0919	62
60%PEG400	0.1156	78
80%PEG400	0.1102	75
100%PEG400	0.1059	72

### Scanning electron microscopy of MSG-CLEAs

**Figure 4:**
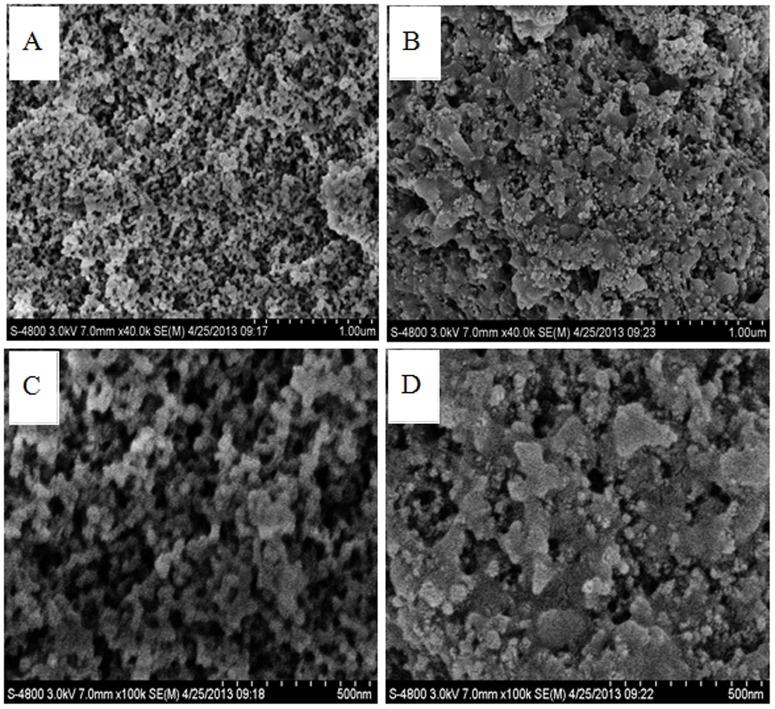
SEM images of (A) modified MSG magnified 40000×; (B) MSG -CLEAs- magnified 40000×; (C) modified MSG magnified 100000×; (D) MSG-CLEAs magnified 100000×.

The SEM image of MSG and MSG-CLEAs were shown in Fig. 4. It could be observed in Fig. 4A that the modified MSG exhibited abundant pores and channels which allow high enzyme immobilization capacities due to high surface areas. As shown in Fig. 4B, CLEAs were formed in these macropores as MSG-CLEAs, and the CLEAs entrapped in the pores would not leak out because the covalent attachments between the MSG and CLEAs molecules formed via cross-linkages with glutaraldehyde. This attachment could be obviously observed in Fig. 4C and 4D that MSG-CLEAs showed rougher surface morphology as a result of cross-linked PAL aggregates on the MSG (Fig. 4D), whereas, the surface of the MSG was very smooth (Fig. 4C).

### Effect of pH and temperature on the free and immobilized MSG-CLEAs

**Figure 5:**
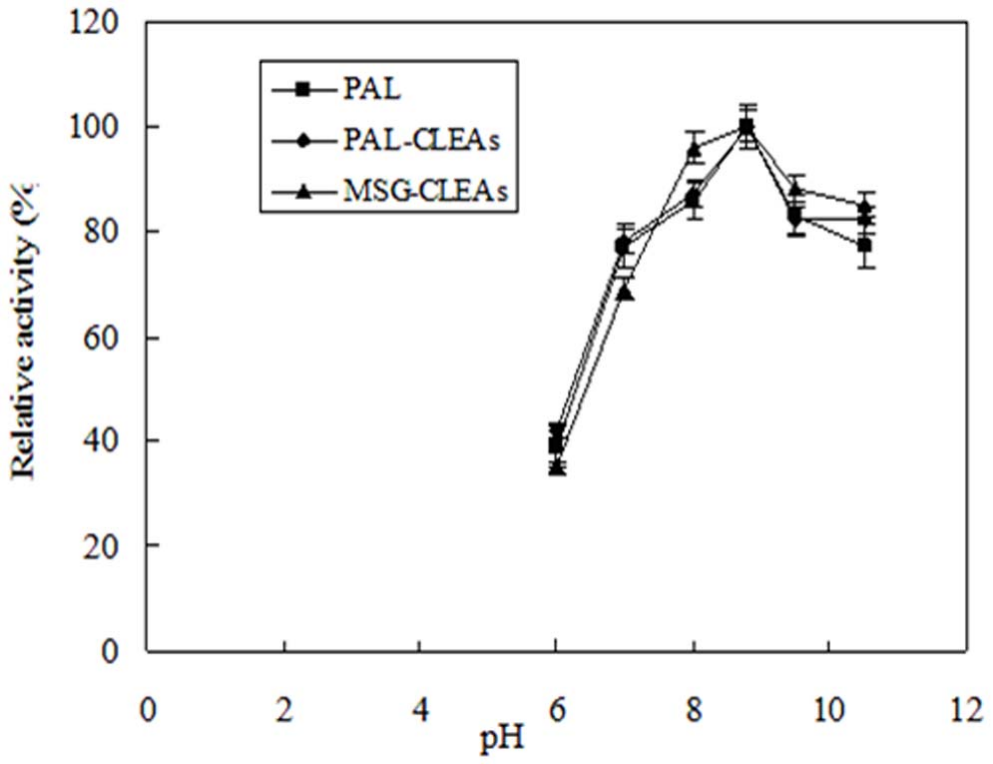
The optimal pH curves of MSG-CLEAs. Enzyme activity was determined according to described in the PAL activity assay section. The enzymatic activity which corresponds to 100% activity is 1.5 U/g and 0.9 U/g wet derivative for the CLEAs and MSG-CLEAs, and 4.5 U/g for the soluble enzyme.

**Figure 6:**
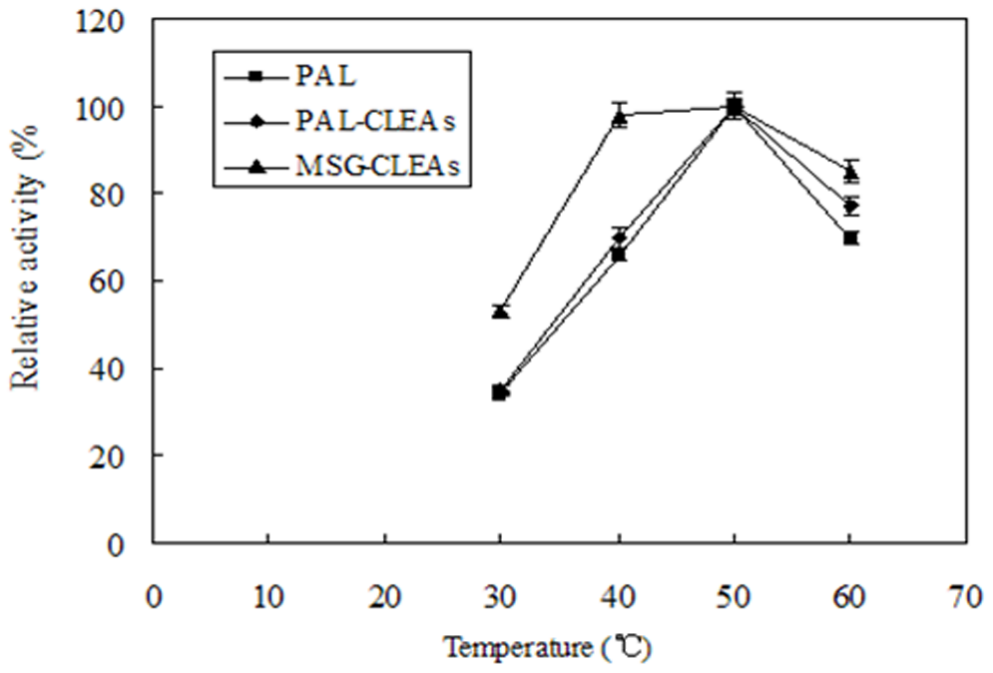
The optimal temperature curves of MSG-CLEAs. Enzyme activity was determined according to described in the PAL activity assay section. The enzymatic activity which corresponds to 100% activity is 0.36 U/g and 0.43 U/g wet derivative for the CLEAs and MSG-CLEAs, and 1.3 U/g for the soluble enzyme.

The effect of pH variations in the range of 6.0–10.5 on the free PAL, PAL-CLEAs and MSG-CLEAs activities was examined at 25°C, as shown in Fig. 5. The results showed that the optimal pH values of all free PAL, PAL-CLEAs and MSG-CLEAs were minor. However, MSG-CLEAs revealed broader enzyme activity compared with free enzyme and PAL-CLEAs. The broader enzyme activity of the MSG-CLEAs perhaps resulted from the stabilization of PAL molecules through the covalent immobilization of MSG-CLEAs on the surface of the MSG. Similar results were obtained from the temperature variance study on the free and immobilized MSG-CLEAs (Fig. 6). MSG-CLEAs did not increase the optimal temperature obviously but extended the optimal temperature range. Moreover, the immobilized preparation gave a significantly broader activity profile than did the free PAL with more heat resistance at high temperatures. The covalent bond formation among enzymes and between CLEAs and MSG might also reduce the conformational flexibility, which stabilize the conformation of active sites of enzyme. Similar results were observed by other groups [7], [26].

### Kinetic parameters

Kinetic parameters of free PAL, PAL-CLEAs and MSG-CLEAs were shown in Table 2. The results showed that the *K*_m_ values of the PAL-CLEAs and MSG-CLEAs obtained were higher than those of the free PAL, which suggest the cross-linked network limited the permeation rate of substrate and product. This result showed that a conformational change due to immobilization resulted in the reduction in affinity of the enzyme to its substrate. On the other hand, *V*_max_ values of PAL-CLEAs and MSG-CLEAs were smaller than that of free PAL. It was indicated that the structure of the enzyme was changed by the immobilization, which resulted in lower accessibility of the substrate to the active sites of the immobilized enzyme [29], [30].

**Table 2:** Kinetic parameters of free PAL, PAL-CLEAs and MSG-CLEAs.

Form of PAL	*K*_m_ (mM)	*V*_max_ (mM/min)
PAL	0.522	0.33
PAL-CLEAs	0.685	0.125
MSG-CLEAs	1.34	0.033

### Stability of the free PAL, PAL-CLEAs, and MSG-CLEAs against chemical denaturants

**Figure 7:**
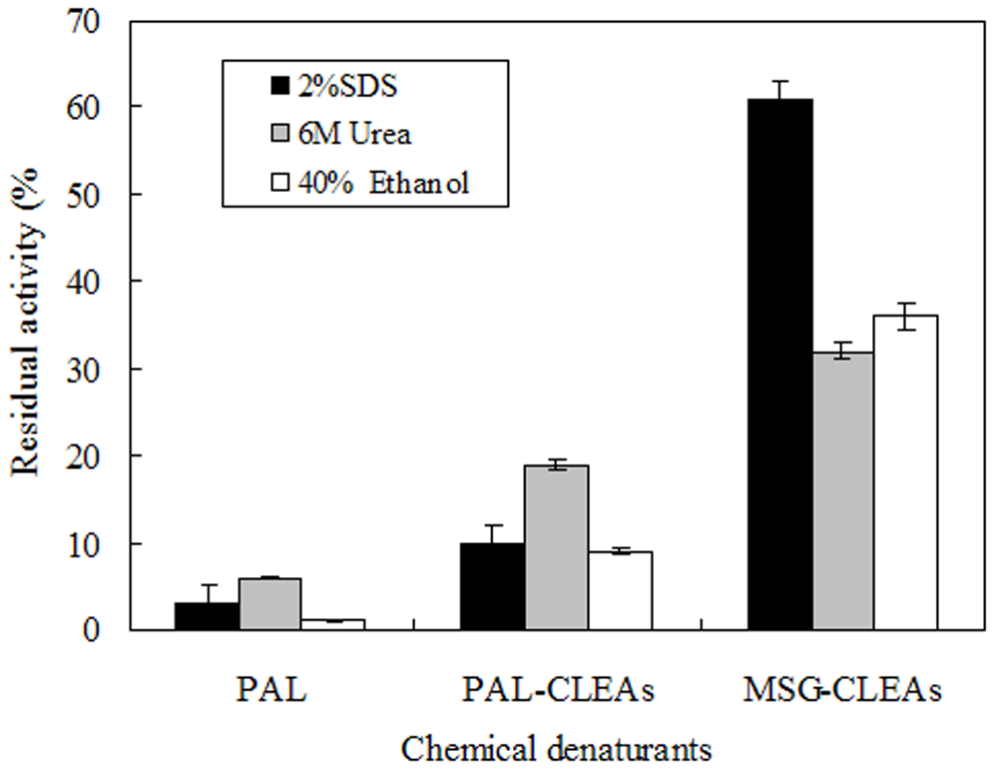
Stability of free-PAL, PAL-CLEAs and MSG-CLEAs against chemical denaturants. Free PAL and immobilized enzyme were stored in urea (6 M), sodium dodecyl sulfate (SDS, 2%, w/v) or ethanol (40%, v/v) in 0.2 M phosphate buffer (pH 7.0) at 30°C for 30 min, respectively. The enzymatic activity which corresponds to 100% activity is 0.36 U/g and 0.43 U/g wet derivative for the CLEAs and MSG-CLEAs, and 1.3 U/g for the soluble enzyme.

As is well known, alcohol, urea and SDS are well known denaturants to proteins. In this study, stability of the free PAL, PAL-CLEAs, and MSG-CLEAs in different chemical denaturants was studied and is shown in Fig. 7. These denaturants inactivated the enzyme in both forms, but the MSG-CLEAs were relatively more tolerant against this inactivation. after being incubated in the presence of 40% alcohol, the free PAL showed almost no activity, while PAL-CLEAs retained 10% of the initial activity and MSG-CLEAs conserved about 35% activity. Furthermore, MSG-CLEAs were relatively more resistant against 6 M urea than free PAL and PAL-CLEAs. Especially, after being incubated with 2% SDS, MSG-CLEAs still showed about 60% of initial activity whereas free PAL and PAL-CLEAs showed only approximately 4% and 10% of initial activity. These results indicated that the covalent cross-linking between the amino groups of the enzyme through glutaraldehyde as well as the covalent inter-cross-linking between CLEAs and MSG surfaces provided more molecular conformation stability of PAL.

### Thermal stabilities of the free PAL, PAL-CLEAs, and MSG-CLEAs

**Figure 8:**
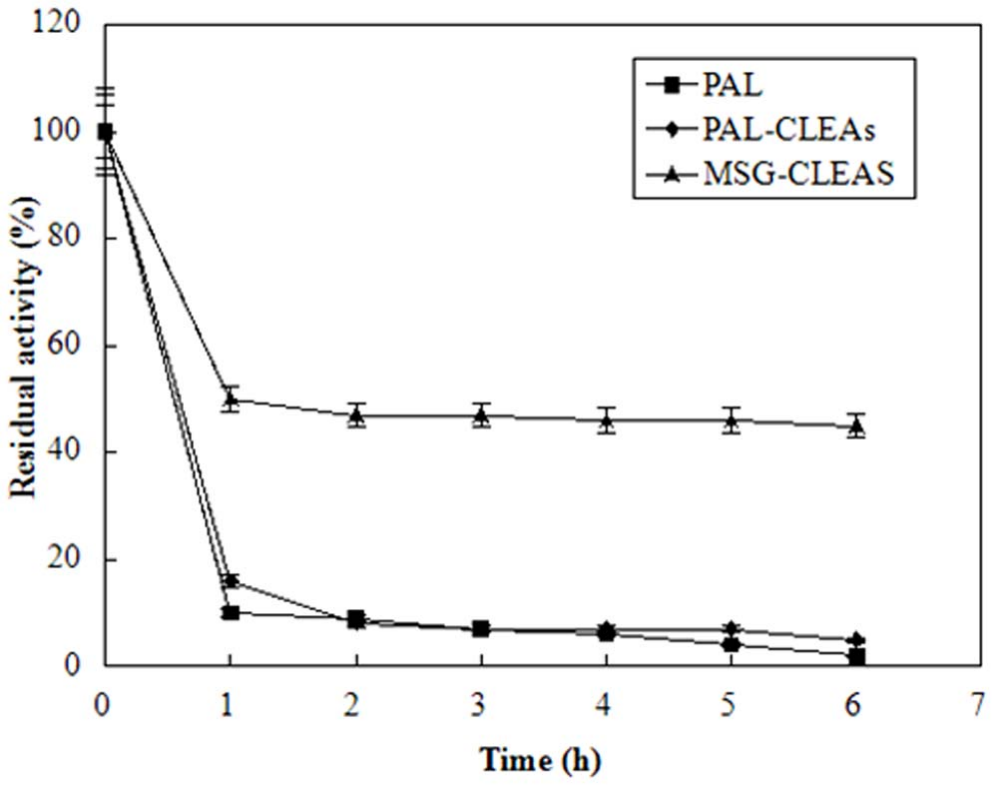
The thermal stability of free-PAL, PAL-CLEAs and MSG-CLEAs at different temperatures. At different incubation times, samples of immobilized enzyme stored in 0.2 M phosphate buffer (pH 7.0) without substrate at 60uC were withdrawn and assayed for activity by described in the experimental section. The enzymatic activity which corresponds to 100% activity is 0.36 U/g and 0.43 U/g wet derivative for the CLEAs and MSG-CLEAs, and 1.3 U/g for the soluble enzyme.

The thermal stability of free and immobilized PAL were showed in Fig. 8. After 6 h incubation, the free PAL showed almost no activity, PAL-CLEAs only retained 7% of the initial activity, while MSG-CLEAs still retained 47% of the initial activity. Compared to the free form, the covalent inter-cross-linking among enzymes showed improvement on its activity to some extent. In contrast, the covalent inter-cross-linking between CLEAs and MSG surfaces as well as the covalent intra-cross-linking among enzyme aggregates improved the rigidity of the active conformation than free enzyme and PAL-CLEAs. As a result, It was more difficult to ruin the conformation of enzyme in MSG-CLEAs than that of free enzyme and PAL-CLEAs under the same thermal condition [7], [28].

### Recyclability of the MSG-CLEAs

**Figure 9:**
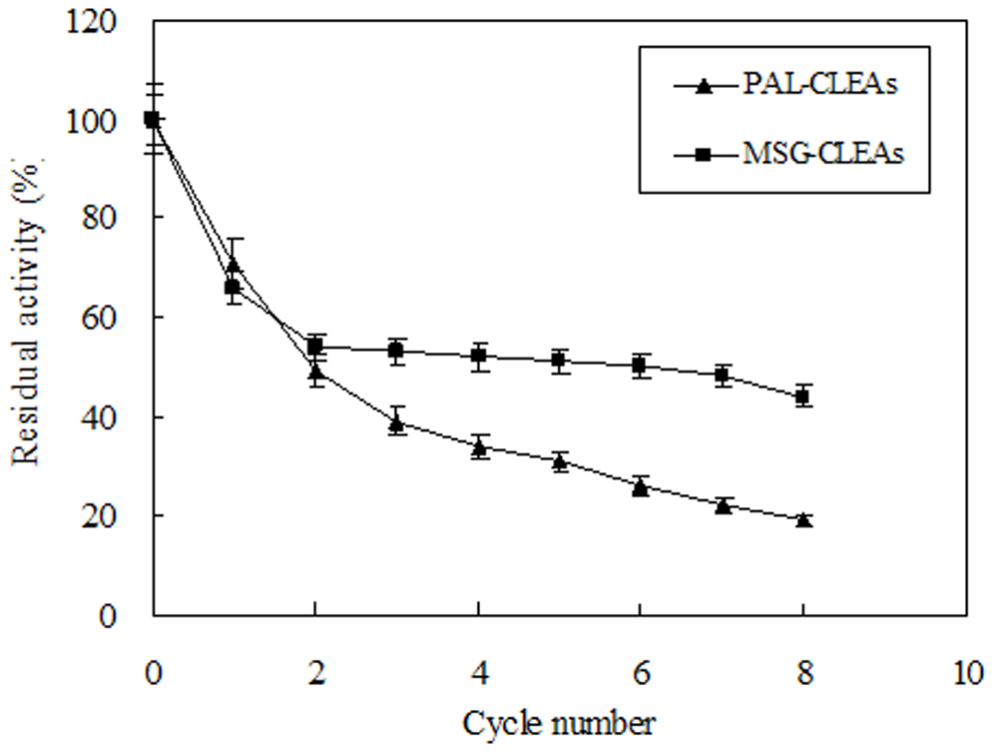
Operational stability of PAL-CLEAs and MSG-CLEAs.

The reusability of immobilized enzymes is important for routine industrial applications [27]. Recyclabilities of PAL-CLEAs and MSG-CLEAs were shown in Fig. 9. Both conventional CLEAs and MSG-CLEAs retained their activities after 8 cycles of reaction although to different degrees. However, after 8^th^ cycle, the conventional PAL-CLEAs only retained about 20% activity whereas MSG-CLEAs still maintained 50% activity. The higher stability of MSG-CLEAs might be due to the fact that the covalent inter-cross-linking between CLEAs and MSG surfaces as well as the covalent intra-cross-linking among enzyme aggregates favors the rigidification of the tertiary structure of the enzyme which creates a more stable preparation than PAL-CLEAs. These results indicated that MSG-CLEAs might be improve the properties of conventional CLEAs in industrial application.

### Storage stabilities of the free, PAL-CLEAs and MSG-CLEA

**Figure 10:**
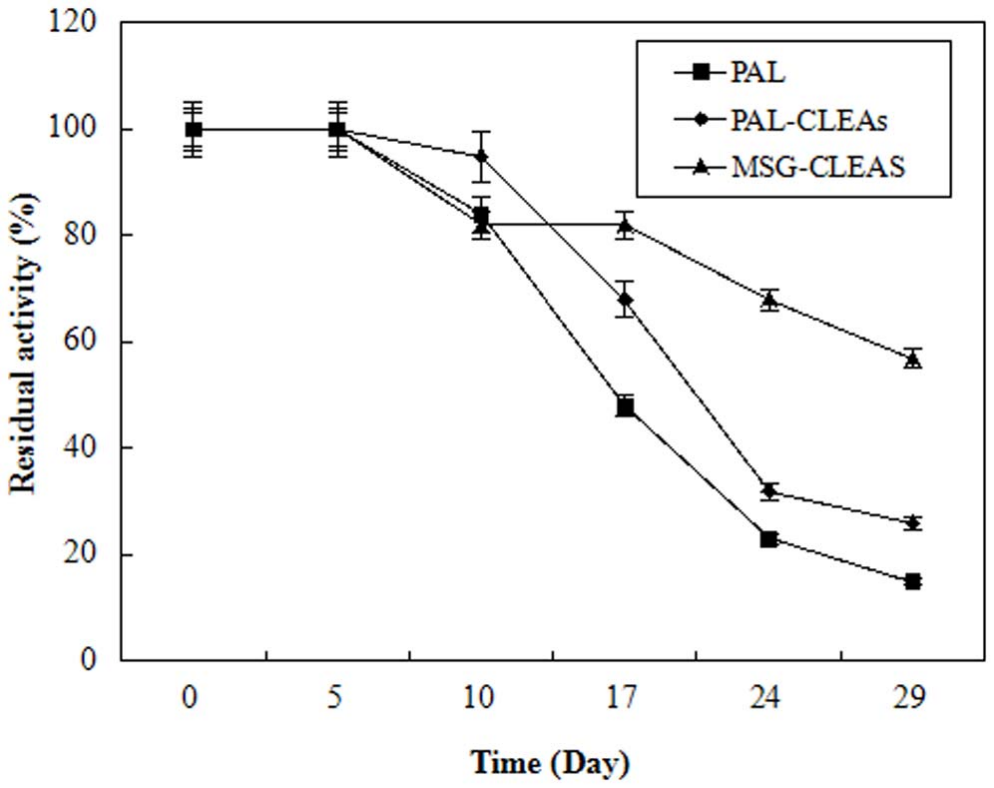
The storage stability of free-PAL, PAL-CLEAs and MSG-CLEAs. At different storage times, samples of immobilized enzyme stored in 0.1 M phosphate buffer (pH 7.0) without substrate at 25°C were withdrawn and assayed for activity by described in the experimental section. The enzymatic activity which corresponds to 100% activity is 0.36 U/g and 0.43 U/g wet derivative for the CLEAs and MSG-CLEAs, and 1.3 U/g for the soluble enzyme.

The storage stability of an enzyme plays an important role in enzyme bioprocesses. In this study, the effect of immobilization on the storage stability of PAL, free enzyme, PAL-CLEAs and MSG-CLEAs was studied. As shown in Fig. 10, the activity of the MSG-CLEAs decreased at a much slower rate than did the free PAL and PAL-CLEAs. Free PAL lost its most activity after 24 days, but PAL-CLEAs and MSG-CLEAs retained about 40% and 70% of their initial activities, respectively after the same storage period. This result showed that the storage stability of PAL was improved considerably after the immobilizing the free PAL by cross-linking of the enzyme in MSG-CLEAs. The more likely explanation was that the multipoint immobilization of MSG-CLEAs prevents the dissociation of enzyme from its matrix and further prevent the leaking of enzyme into aqueous buffer [7], [28].
